# The marketing firm and consumer choice: implications of bilateral contingency for levels of analysis in organizational neuroscience

**DOI:** 10.3389/fnhum.2014.00472

**Published:** 2014-07-02

**Authors:** Gordon R. Foxall

**Affiliations:** Cardiff Business School, Cardiff UniversityCardiff, UK

**Keywords:** consumer behavior analysis, behavioral perspective model, marketing firm, bilateral contingency, emotion, neuroeconomics, levels of explanation, organizational neuroscience

## Abstract

The emergence of a conception of the marketing firm (Foxall, 1999a) conceived within behavioral psychology and based on a corresponding model of consumer choice, (Foxall, 1990/2004) permits an assessment of the levels of behavioral and organizational analysis amenable to neuroscientific examination. This paper explores the ways in which the bilateral contingencies that link the marketing firm with its consumerate allow appropriate levels of organizational neuroscientific analysis to be specified. Having described the concept of the marketing firm and the model of consumer behavior on which it is based, the paper analyzes bilateral contingencies at the levels of (i) market exchange, (ii) emotional reward, and (iii) neuroeconomics. Market exchange emerges as a level of analysis that lends itself predominantly to the explanation of firm—consumerate interactions in terms of the super-personal level of reinforcing and punishing contingencies: the marketing firm can be treated as a contextual or operant system in its own right. However, the emotional reward and neuroeconomic levels of analysis should be confined to the personal level of analysis represented by individual managers on the one hand and individual consumers on the other. This also entails a level of abstraction but it is one that can be satisfactorily handled in terms of the concept of bilateral contingency.

## Introduction

### Levels of analysis in organizational neuroscience

An important issue for the emergent discipline of organizational neuroscience is to determine the levels of analysis at which its explanations of behavior may be properly directed. Four such levels may be proposed as appropriate to the explanation of behavior in terms of neurophysiological and environmental (reinforcing and punishing) events: the *sub-personal* level of exposition refers to neurophysiological events; the *personal* level, to the beliefs, desires and other intentional idioms that are ascribed to the individual to account for his/her behavior; the *super-personal* level to the environmental influences that shape and maintain behavior (i.e., reinforcers and punishers); and the *supra-personal* level to the emergent behavior of an organization such as the firm.

Any explanation of behavior in terms of the sub-personal level of neuronal activity (Dennett, 1969), enjoins methodological individualism as a philosophy of science on its practitioners. After all, the neurophysiology of an individual can enter into the explanation of the behavior of that person alone. However, while the behavioral analysis of individual members of organizations proceeds well enough in neurophysiological terms, it is sometimes necessary to understand and predict the behavior of the organization as a whole. Even explanations of behavior based on radical behaviorist models have recently embraced the idea that an organization might be treated as a contextual or operant system in its own right, its behavior predictable from those of its outputs that are over and above the joint consequences of the behaviors of its members (Glenn, 1991, 2004; Foxall, 1999a; Glenn and Malott, 2004; Biglan and Glenn, 2013). How far is it feasible to construct such an account of organizational behavior on the basis of neurophysiological knowledge?

This may constitute an abstraction too far for traditional behavior analysts for whom the individual organism is the sole bearer of behavior that is to be environmentally explained; but at least the behavioral outputs of supra-individual entities such as organizations are identifiable by intersubjective agreement. The same cannot be said of sub-personal events which are employed in organizational neuroscience to explain the behavior of individual managers; although the effects of such events may be demonstrated under highly-restrictive laboratory conditions, their application in the interpretation of the complex behaviors that characterize human interactions in organizations requires some ground rules for the explanation of personal level behavior by means of inferred sub-personal occurrences.

Since the marketing firm is conceptualized as an organization whose existence is closely tied up with the satisfaction of consumer wants, the analysis of consumer behavior is a prerequisite of the corporate-level investigation appropriate to the marketing firm. The behavior of consumers is depicted comparatively easily in neuroscientific terms because each consumer can be treated as an individual; this enables analysis to embrace the personal level of analysis which harmonizes with the possibility that sub-personal (neuronal) events within the organism may play a causal role in explaining the organism's behavior. When we consider the behavior of an individual in terms of the super-personal causal texture provided by the consequences of behavior, that is when we consider the individual to be a contextual or operant system, we can specify once again how the persistence of this behavior is influenced by the reinforcing and punishing outcomes it produces. A recent extension of this idea is that the behavior of organizations can be predicted and explained by considering them in their entirety as “contextual systems.” A contextual system is an entity the behavior of which can be predicted and explained by reference to its learning history and its current behavior setting; that is by the consequences that have followed its behaviors in the past as they interact with current opportunities to repeat similar behaviors or to engage in competing activities (Foxall, 1999b). This basic assumption of the concept of the marketing firm (Foxall, 1999a), has also been incorporated into behavior analytic thinking through the analysis of metacontingencies (e.g., Biglan and Glenn, 2013).

A complication arises, however, if we seek to apply neuroscientific thinking to the behavior of a supra-personal entity such as a firm or other organization. There is no analog in the organization of the neuronal firing in terms of which individual behavior can be construed. It is, therefore, necessary to deal not with the organization as a neurological unit but with the individual members of the organization whose behavior may be understood by reference to the behavior of other organizational members or external actors. This paper is concerned, nevertheless, to explore the implications of this in order to assess the contribution of organizational neuroscience to the explanation not only of individual managers and consumers, but to the interactions of the marketing firm and its consumerate^1^. The key to this lies in the *bilateral contingencies* that these interactions create and maintain (Foxall, 2014a).

### Consumer behavior and marketing management

Although marketing management is generally understood as a response to the demands of the marketplace, it is unusual for a theory of managerial marketing to proceed in similar terms to those in which the underlying theory of consumer choice is couched. The research program encompassing the Behavioral Perspective Model of consumer behavior (BPM: Foxall, 1990/2004, 2013) and the Theory of the Marketing Firm (TMF: Foxall, 1999a), which employ interfacing operant models, attempts to address this inconsistency. Both models and the interactions they posit have received empirical support in research that has focused on the behavior of the marketing firm as a whole in relation to other firms in the market ^2^.

This paper proposes and explores a level of analysis that has not previously featured in studies of the marketing firm, namely the neuropsychological and neuroeconomic implications of the completion of successful exchanges with the firm's consumers. The emerging discipline of organizational neuroscience (e.g., Butler and Senior, 2007a,b; Lee and Chamberlain, 2007; Lee et al., 2007; Becker and Cropanzano, 2010) provides a general framework for this analysis, which is extended by the incorporation of some aspects of neuroeconomics to capture the economic and social exchange relationships that characterize marketer-consumer relationships. While the behavior of consumers has been explicated in terms of its neurophysiological underpinnings (Foxall, 2008, 2011), those of managerial and non-managerial firm members have not yet been characterized in this way. The BPM/TMF framework proceeds in terms of operant psychology and operant behavioral economics and it is within this disciplinary matrix that the present paper is constructed. However, the close relationship between reinforcement learning and the operation of the dopaminergic reward prediction error (RPE) system provides an additional reason for undertaking a neuroeconomic analysis of behavior in operant terms (Stanton et al., 2010; Caplin and Glimcher, 2013; Daw, 2013; Daw and Tobler, 2013). This paper therefore examines the activities of mangers conceived in operant terms. Although the paper focuses on managerial rather than non-managerial organizational behavior, motivation of the latter is implicit in its treatment of the former since a central component of managerial behavior involves the management of other members of the firm whose motivation must be taken into account.

The paper describes the BPM and TMF approaches before examining in greater detail than hitherto the nature of the bilateral contingencies that link consumer behavior and marketing management. Three levels of analysis of bilateral contingencies are proposed, referring respectively to market-exchange relationships, emotional rewards, and neuroeconomics interactions. The concluding section discusses the capacity of organizational neuroscience to employ analyses of this kind.

## Consumer behavior analysis

The BPM (Foxall, 1990/2004) is an elaboration of the “three-term contingency,” the basis explanatory device of operant behaviorism. In the three-term contingency, a consequential stimulus influences the rate at which a previously-emitted response is repeated (reinforced); any antecedent stimulus present when reinforcement takes place may come to exert control over the subsequent emission of the response, even in the absence of the reinforcer. In summary,

$${\text{S}}^{\text{D}}\to \text{R}\to {\text{S}}^{\text{R}}$$

where S^D^ is a *discriminative stimulus*, i.e., an element of the environment in the presence of which an organism performs selectively by emitting a response, R, which has previously been reinforced in the presence of the S^D^; and S^R^ is the reinforcing stimulus ^3^.

The efficacy of a learning history is thus understood as the way in which the outcomes of prior behavior influence current choice. In recent years a 4-term contingency has been proposed in which a motivating operation (MO) is an antecedent event that enhances the relationship between the response and the reinforcer (Michael, 1982; for conceptual and empirical development in the context of consumer behavior analysis, see Fagerstrøm, 2010; Fagerstrøm et al., 2010). An advertisement that promises “This product will stimulate your taste buds like nothing you've ever experienced!” is an example of a MO.

**Figure 1:**
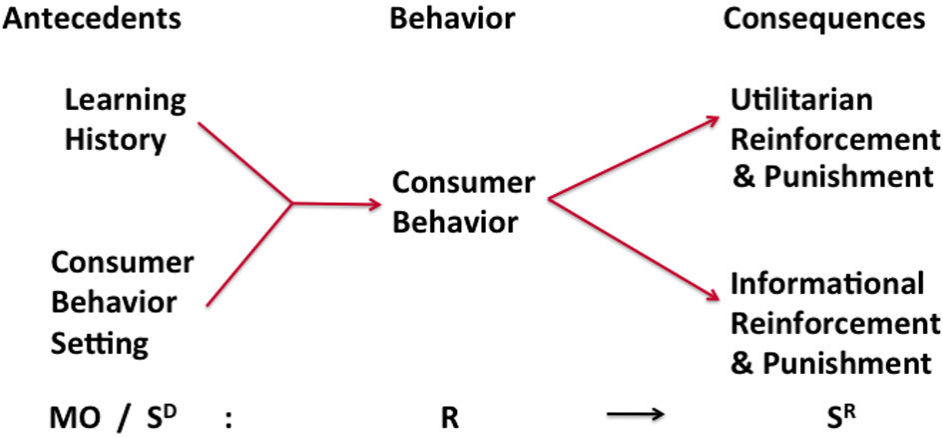
**The behavioral perspective model of consumer choice**. Adapted from Foxall (1990/2004). Used by permission.

This basic paradigm is elaborated in the BPM to bring it into service as a means of predicting and interpreting human economic behavior in naturally-occurring settings. In the BPM, the immediate precursor of consumer behavior is the *consumer situation* which represents the interaction of the consumer's learning history and the discriminative stimuli and MOs that make up the current behavior setting (Figure 1). In this interaction, the consumer's experience in similar contexts primes the setting stimuli so that certain behaviors are made more probable while others are inhibited. Consumer behaviors that are encouraged by the consumer situation are those that have met with rewarding or reinforcing consequences on previous consumption occasions while those that are discouraged are those that have been punished. The consequences of consumer behavior, i.e., its reinforcing and aversive outcomes, are of two kinds: *utilitarian reinforcement and punishment* consists in the behavioral consequences that are functionally related to obtaining, owning and using an economic product or service, while *informational reinforcement and punishment* stem from the social and symbolic outcomes of consumption. Consumer behavior is therefore a function of the variables that make up the current consumer behavior setting insofar as these prefigure positive and aversive utilitarian and informational consequences of behaving in particular ways. A more closed consumer behavior setting is one in which one or at most a few behaviors are available to the consumer, while a more open setting is one which presents the consumer with a multiplicity of ways of acting. The topography of consumer behavior is then predictable from the pattern of utilitarian and informational reinforcement which the setting variables signal to be available contingent on the enactment of specific consumer behaviors.

**Figure 2:**
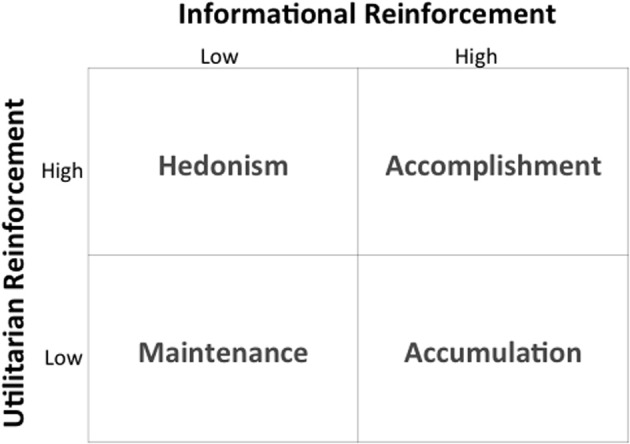
**Pattern of reinforcement and operant classes of consumer behavior**. Adapted from Foxall (1990/2004). Used by permission.

**Figure 3:**
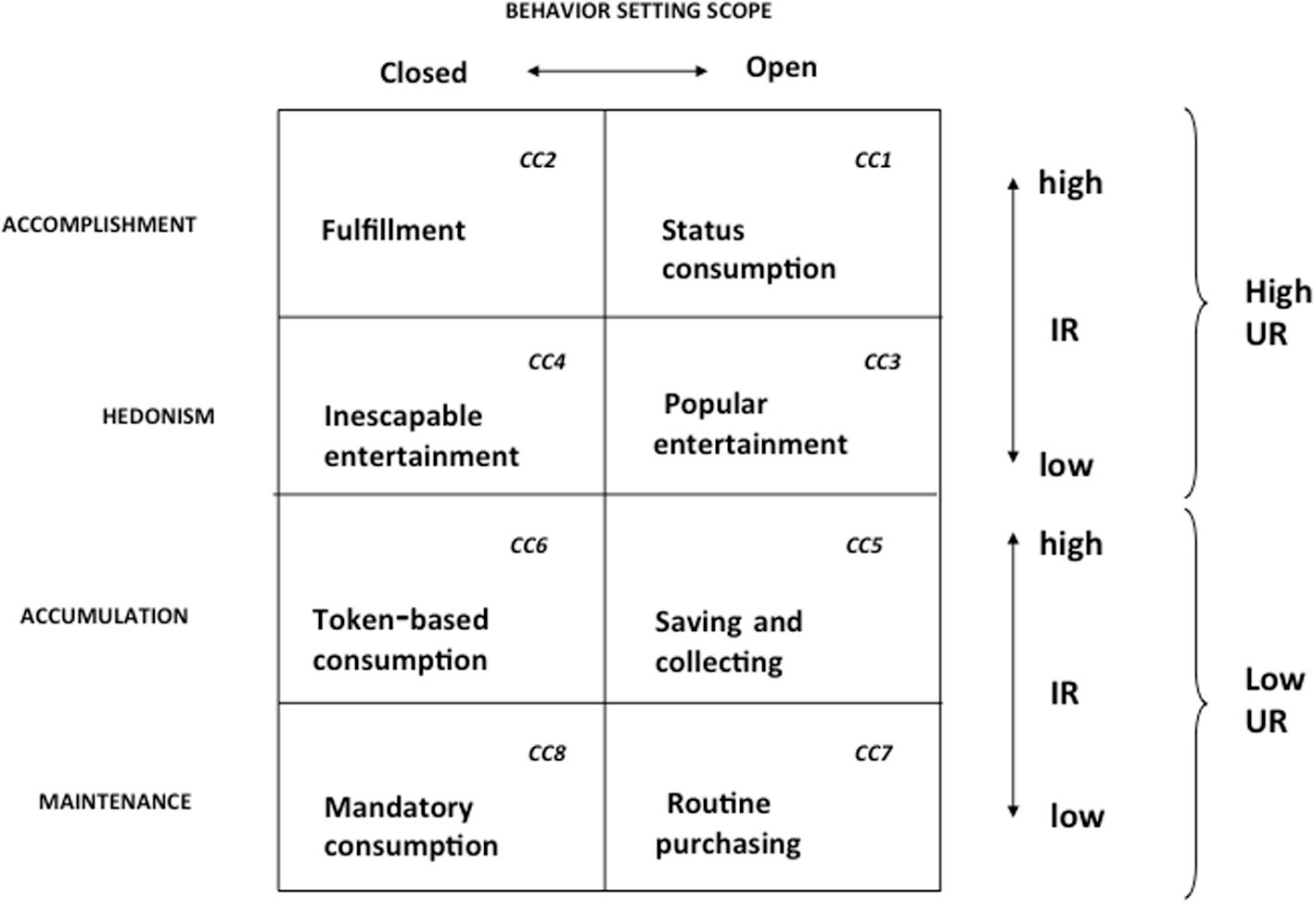
**The BPM contingency matrix**. Adapted from Foxall (1990/2004). Used by permission. CC, Contingency Category.

Figure 2 shows the patterns of reinforcement that maintain consumer choice, along with the operant classes of consumer behavior that they define. Figure 3, the BPM Contingency Matrix, further incorporates the scope of the consumer behavior setting to provide a functional typology of the contingency categories defined by the model. (For a full exposition of the model, see Foxall, 2010). Empirical research demonstrates that changes in consumer behavior, measured as elasticity of demand for fast moving nondurables is a function of the pattern of utilitarian and informational reinforcement (Foxall et al., 2004, 2013; Oliveira-Castro et al., 2011; Yan et al., 2012a,b); moreover, consumers' utility functions can be estimated to demonstrate that they maximize measurable combinations of these goods: Oliveira-Castro et al. (under review) show that consumers maximize selected combinations of utilitarian reinforcement and informational reinforcement as depicted by the following Cobb-Douglas utility function:

(1) $${\text{U}}_{\left(\text{x1},\text{x2}\right)}={\text{x}}_{1}^{\text{a}},{\text{x}}_{2}^{\text{b}}$$

where U is the total amount of utility obtained by consumption of x_1_ and x_2_, x1 is the quantity of utilitarian reinforcement consumed, x_2_ is the quantity of informational reinforcement consumed, and a and b are empirically determined parameters such that a + b = 1. Furthermore, empirical research suggests that consumers ultimately maximize a combination of emotional responses to consumption situations (Foxall, 2011; Foxall et al., 2012). In short, we now have a clear picture of the reward structure that shapes and maintains consumer choice, the neurophysiological processes that govern this structure, and the nature of the emotional utility function which consumers optimize.

## The marketing firm

The underlying premise of the marketing firm concept (TMF; Foxall, 1999a) is that firms exist in order to market within the competitive structures that compel firms to adopt customer-oriented marketing as a general managerial philosophy is they are to survive (avoid loss) and prosper (innovate in ways that encourage a satisfactory level of sales. The concept reflects elements of the thought of Coase (1937), Simon (1976), and Drucker (2007). The structural conditions that compel such marketing-orientation are marked by the ability to productive capacity to generate supply that exceeds demand, the existence of large levels of discretionary income on the part of consumers engendering inter-industrial competition among firms, and a sophisticated consumerate, i.e., buyers who are knowledgeable with respect to the products they purchase and the alternative offerings available in the marketplace (Foxall, 1981).

The resulting framework of conceptualization and analysis understands corporate institutions as organized patterns of behavior maintained by their consequences, namely the rewards and sanctions that follow them (or, more accurately and avoiding teleology, that have followed them in the past). The behavior of the marketing firm eventuates in the introduction of marketing mixes that offer product, price, promotion, and place utilities to consumers (Foxall, 1999a). The success of the firm, hence its future behavior, depends on the reception these marketing mixes receive in the marketplace. This perspective, based on selection by consequences (Skinner, 1981), permits continuity with evolutionary theories of the firm (Hodgson and Knudsen, 2010) by embracing the same explanatory principles of selection by consequences that underlies Darwinian natural selection but extending it to events in the ontogenetic development of individuals and organizations. Van Parijs (1981) refers to these explanation as N-evolution and R-evolution respectively, noting the role of natural selection (N) in the former and of reinforcement (R) in the latter.

More specifically, the concept of the marketing firm portrays corporate behavior in marketing-oriented enterprises as the management of the scope of the consumer behavior setting and the pattern of reinforcement available to the consumer. The relationship of the firm and its consumers is depicted in terms of bilateral contingencies in which the behavior of marketers in reinforced and punished by consumer behaviors while consumer behavior is reinforced and punished by managerial actions. This paper concentrates on these *marketing relationships* that are characterized by tangible exchanges of property rights between the firm and its consumers. Its purpose is to complete the picture of *bilateral contingency* between the firm and its consumers by probing (i) what are the reward structures of managers within the marketing firm? (ii) how are these underpinned by neurophysiological processes? (iii) the nature of managers' utility functions. The TMF framework also draws a distinction between two kinds of relationship. The first, between the firm and its customers, between principal and agent within the organization, between the firm and its suppliers, all of which entail literal exchange of legal rights are known as “marketing relationships.” Other relationships that do not proceed on this basis even though they may be essential to forming and maintaining marketing relationships, such as social and trade association contacts among firms and broader noncontractual relationships between managers and other employees, are known as “mutuality relationships” (Foxall, 1999a; Vella and Foxall, 2011). This paper is concerned primarily with the former.

The behavior of managers within the marketing firm exhibits many similarities with intra-firm managerial behavior generally. These managerial behaviors have been a central concern of organizational neuroscience. There is a need for cooperation with other managers and other employees, for instance. Work which examines the neurophysiological basis of trust (Zak, 2004, 2007; Zak and Nadler, 2010), cooperation and conflict (Levine, 2007; Tabibnia and Lieberman, 2007), and social interaction (Caldú and Dreher, 2007) are of special interest in the analysis of both mutuality and exchange/marketing relationships. This is especially pertinent to the management of mutuality relationships within the firm as well as outwith the organization, say between the firm and its suppliers. The neurophysiological basis of behavior is not likely to differ among managers but the sources of the rewards they undertake will uniquely follow the pattern of responsibilities their separate job descriptions entail. The various types of decision, from the most administrative or programmed to the most strategic and unprogrammed, that each of these responsibilities requires will have implications for the kind of neurophysiological functioning we can infer (Foxall, 2014b). It is to the strategic sphere, management of bilateral relationships, those that span the connections between the firm and its various publics, that this paper pays special attention, for the very nature of marketing management and the activities of the marketing firm are defined and oriented toward such interactions.

The present analysis is concerned principally with the neurophysiological implications of managerial behavior insofar as it is influenced by the bilateral relationships between the firm and its consumers. Specifically, it traces the sources of reward that sustain these relationships for individual managers. Bilateral contingency implies that the behavior of managers is reinforced by the outcomes of consumer behavior just as consumer behavior is reinforced by the outputs of the marketing firm in the form of products and services. The emphasis is therefore on the *marketing relationships*, those that entail literal exchange, between an executive engaged in marketing management within a supplier organization and its consumers.

## Bilateral contingency

### The nature of bilateral contingency

Behavior analysts have traditionally adopted the individual organism as their unit of analysis. However, by treating the organization that is the marketing firm as a contextual or operant system in its own right, and by assuming that the function of such a firm is to pursue marketing- or customer-oriented marketing, it becomes feasible to interpret the behavior of its mangers in terms of the context provided by its customers.

The relationships between the marketing firm and its customers can be conceptualized in terms of bilateral contingencies (Foxall, 1999a). The essence of this approach is that the behavior of an organization is greater than/different from that of the combined repertoires of its members. This conception, which has always been integral to the concept of the marketing firm, is supported by recent thinking in organizational behavior analysis which envisions the behaviors of organizational members as enmeshed in *interlocking behavioral contingencies* (Glenn, 2004; Biglan and Glenn, 2013). In both systems of thought, the behavior of the system is inferred from the outputs it produces. Hence, each element of the marketing mix—product, price, the promotional communications and distribution systems—affects consumer behavior in such a way as to make the behavior of the organization predictable and explicable. To adopt this kind of analysis is to consider the behavior of an organization or other collectivity of individuals in operant terms, to understand it as a contextual system.

Consideration of the marketing firm as a contextual system has hitherto been confined to the behavioral analysis, in terms of utilitarian and informational reinforcement, of the relationship between its behavioral outputs and their reception by the market and to the scope of the behavior settings of the firm and its customers (Vella and Foxall, 2011, 2013). This has entailed the description and explanation of the firm's behavior in terms of operational measures of behavioral consequences and behavior setting. This is “Market-Exchange Analysis” which is briefly described below. It is feasible, however, to extend the analysis of the marketing firm as a contextual system by comprehending marketer and customer behaviors in neurophysiological terms. This is pursued below in terms of two further analyses: that of the emotional rewards received by consumers and firms as a result of their mutual interactions (“Affective-Reward Analysis”), and that of the capacity of the signals each party to the transaction receives from the other as RPEs that influence its own behavior (“Neuroeconomic Analysis.”)

### Market-exchange analysis

**Figure 4:**
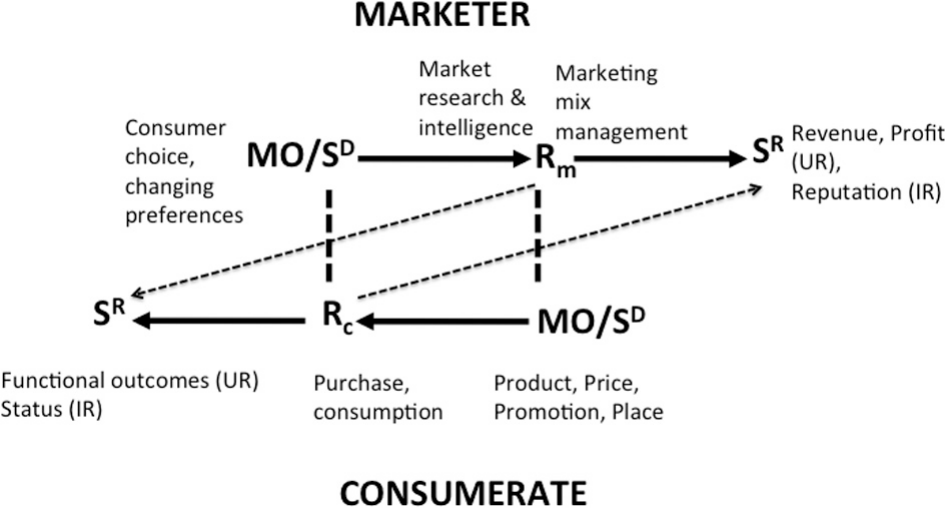
**Bilateral Contingency between the Marketing Firm (the Marketer) and the its Consumerate**.

Market-exchange analysis concerns the overt relationships between the marketing firm and its customer base (Figure 4). The task of marketing management is to plan, devise and implement marketing mixes that deliver satisfactions for the firm's customer base that are profitable for the firm. The components of the marketing mix (product, price, promotion, and place utilities) appear in the market place initially as MOs and discriminative stimuli for the consumer behaviors of browsing, purchasing, and consuming. Purchasing includes the exchange of money for the ownership of the legal right to a product or service and this pecuniary exchange acts as a source of both utilitarian reinforcement (in the form of resources that can be paid out or reinvested) and informational reinforcement (in the form of feedback on corporate performance) for the marketing firm. The efficacy of Rm (managerial behavior) in fulfilling the professional requirements of marketing management, namely the creation of a customer who purchases the product at a price level sufficient to meet the goals of the firm, is determined by the generation of profit and reputation for the firm (depicted by the dotted diagonal line in Figure 4). This consumer behavior (Rc) also acts as MOs and discriminative stimuli for further marketing intelligence activities, marketing planning and the devising and implementation of marketing mixes that respond to the stabilities and/or dynamic nature of the behavior of the customer base (Vella and Foxall, 2011, 2013; Foxall, 2014a).

At this level of market interaction between the enterprise and its customer base, managerial behavior can be viewed as maximizing a utility function of the form shown for the individual consumer in Equation (1), comprising a combination of utilitarian reinforcement and informational reinforcement.

### Affect-reward analysis

**Figure 5:**
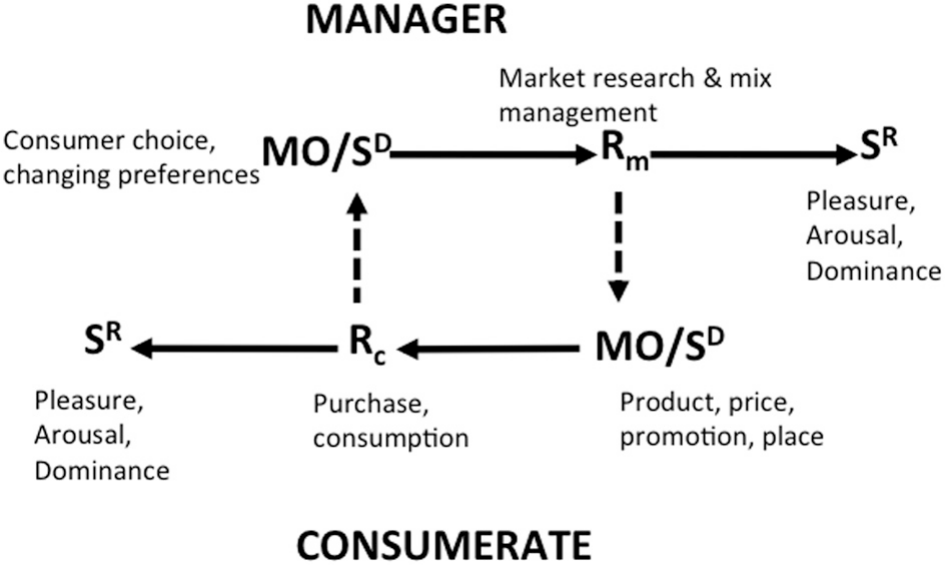
**Bilateral contingency between a manager within the marketing firm and the firm's consumerate in terms of emotional response**.

The second analysis of bilateral contingency is that which exists between individual managers in the marketing firm pursuing marketing-oriented management, as a strategy of the entire enterprise, via marketing management, the responsibility of the marketing function, and their consumers (Figure 5). The relationships between manager and consumer are maintained at this level of analysis by the reciprocal generation of emotional rewards or satisfactions, particularly *pleasure, arousal*, and *dominance* (Mehrabian and Russell, 1974; see also Foxall, 2005).

**Figure 6:**
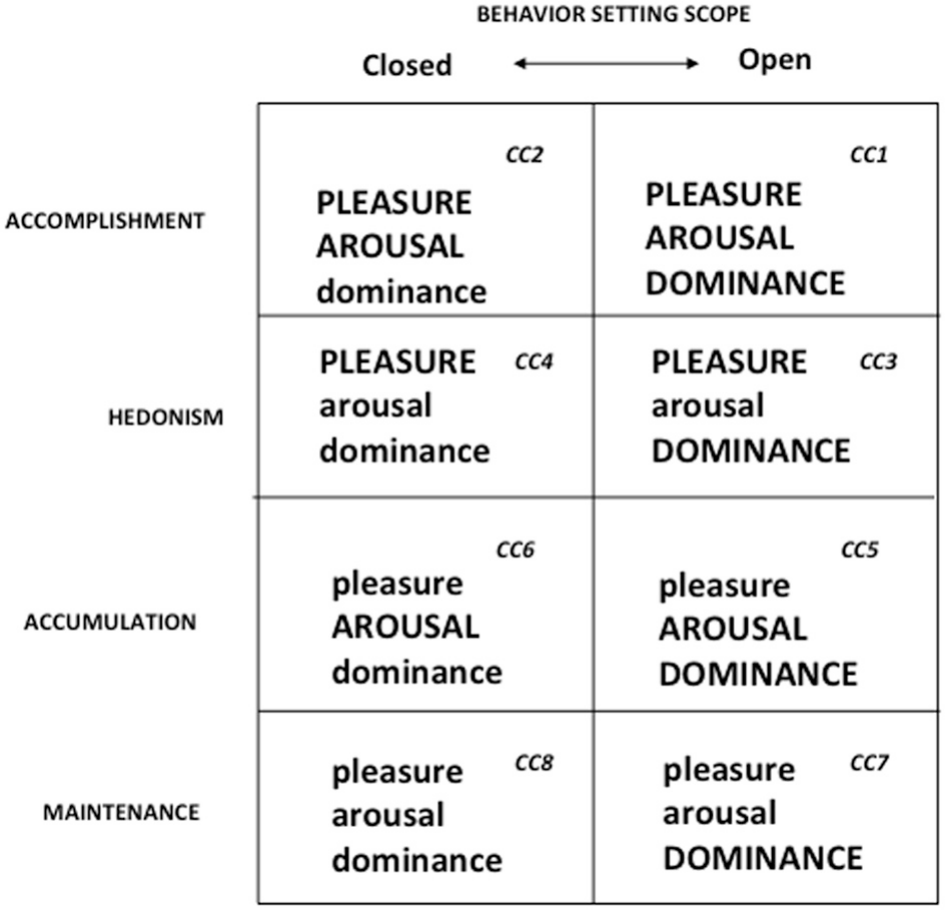
**The BPM emotion-contingency matrix**. *Source*: Foxall (2011). Used by permission. The figure summarizes the research hypotheses and findings. Studies show that: (i) pleasure scores for contingency categories (CCs) 1, 2, 3, and 4 each exceed those of CCs 5, 6, 7, and 8; (ii) arousal scores for CCs 1, 2, 5, and 6 each exceed those of CCs 3, 4, 7, and 8; (iii) dominance scores for CCs 1, 3, 5, and 7 each exceed those for CCs 2, 4, 6, and 8. Moreover, (iv) approach–avoidance (aminusa) scores for CCs 1, 2, 3, and 4 each exceed those for CCs 5, 6, 7, and 8; and (v) approach–avoidance scores for CCs 1 and 3 each exceed those for CCs 2, 4, 5, 6, 7, and 8. (For explication, see text and Foxall et al., 2012).

This hypothesis is supported by the theoretical demonstration of relationships between felt emotion and operant learning as well as by empirical work, albeit with consumers, that shows patterns of emotion to vary consistently and predictably with patterns of reinforcement as defined by the BPM. At the theoretical level, Rolls (1999) suggests a link between learning and emotional reward by proposing that the stimuli that act as reinforcers for behavior also function as elicitors of emotional responses. At the empirical level, there is extensive evidence that consumers respond to retail and consumption environments rich in utilitarian reinforcement with pleasure; to those rich in informational reinforcement with arousal; and to more open settings in terms of dominance. Moreover, consumer behaviors for a wide range of such environments (including the time and money consumers spend within them) has been shown to be determined by these three emotional responses (Foxall, 2011; Foxall et al., 2012; Yani-de-Soriano et al., 2013). Figure 6 summarizes the results of research that indicates a unique pattern of emotional reaction is found for each of the eight BPM-defined contingency categories. We may reasonably conjecture that the responses of individual managers to the reward environments they encounter as members of marketing firms can also be construed in terms of pleasure (derived from utilitarian reinforcement), arousal (informational reinforcement) and dominance (open settings). Although we cannot base this assumption on direct empirical research as is the case for consumer behavior, Mehrabian's theory of emotional responses to environmental cues (Mehrabian, 1980) provides a general warrant for drawing the general conclusion that individual managers' emotional reactions to their reward environments are emotionally reinforced.

**Figure 7:**
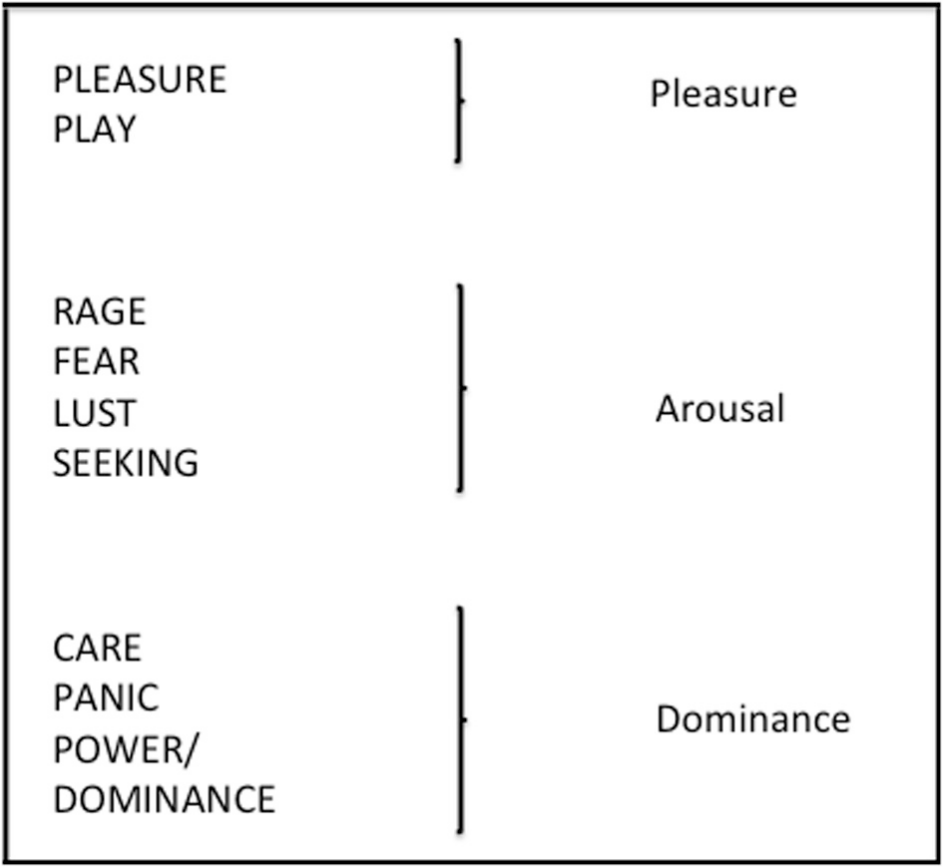
**Panksepp's (1998) seven core emotional systems, augmented by Pleasure and Power Dominance (after Toronchuk and Ellis, 2010) and related to Mehrabian and Russell's (1974) tripartite classification of emotions**.

Regarding pleasure, arousal, and dominance as primary adaptations, it should be possible to identify their neural substrates, their evolutionary significance and their implication in adaptive behaviors (Mehrabian, 1980). Barrett et al. (2007) confirm Mehrabian and Russell's (1974) judgment that pleasure, arousal, and dominance are fundamental to the mental representation of emotion and relate them to reinforcement and punishment (see also Russell and Barrett, 1999; Barrett, 2005; Kober et al., 2008; Lindquist et al., 2012). Moreover, Panksepp's (1998, 2005, 2007) seven core emotional systems – SEEKING, RAGE, FEAR, LUST, CARE, PANIC, PLAY—correspond at a general level to pleasure, arousal and dominance (Foxall, 2008). Figure 7 proposes a broader classification which incorporates PLEASURE and POWER/DOMINANCE following the suggestion of Toronchuk and Ellis (2010).

#### Neurophysiological bases of pleasure, arousal, and dominance

Feelings of *pleasure* are closely related to the evolutionarily-based outcomes of biological fitness; moreover, utilitarian or functional reward promotes the restoration and maintenance of homeostasis (Panksepp, 1998). Expectation of pleasure also facilitates goal-orientation by contributing to the setting of objectives (Politser, 2008). The association of the core emotion of pleasure-displeasure is associated with the utility/disutility of behavioral consequences (Barrett et al., 2007) resulting from approach/avoidance of specific stimuli. This accords with Rolls's (1999) argument that the stimuli that reinforce/punish behavior evoke emotional feeling. Genetic endowment specifies not particular behaviors but the goals of classes of behavior by selecting the stimuli that will reinforce or punish approach and avoidance (Rolls, 2005).

The allocation of localized brain regions to the production of emotions is dangerous since the neuronal basis of any particular source of affect may be distributed (Uttal, 2001; Legrenzi and Umità, 2011; Lindquist et al., 2012). However, there is evidence that self-reports of pleasure coincide with increased activity in the amygdala, orbito-frontal cortex (OFC), and ventromedial prefrontal cortex (vmPFC) (Cardinal et al., 2002; Rolls et al., 2009). Increases in the activation of the ventral tegmental area (VTA), the subcortical telencephalon areas nucleus accumbens (NAC), and parts of the ventral striatum (vStr), all well-endowed with dopaminergic neurons, are associated with pleasant experiences; these correlate too with hypothalamus (Hy), vmPFC, and right OFC activation (Wager et al., 2008). The NAC is closely related to reinforcement and pleasure. Winkielman et al.(2005 p. 346) note that “The nucleus accumbens, which lies at the front of the subcortical forebrain and is rich in dopamine and opioid neurotransmitters, is as famous for positive affective states as the amygdala is for fearful ones.” While defending the role of NAC in positive affect, Berridge and Robinson (1998) maintain that the NAC is implicated in “wanting” a stimulus (known as its incentive salience) rather than “pleasure” in obtaining or consuming it.

Moreover, brain areas closely associated with pleasure-displeasure comprise a region “that is involved in establishing the threat or reward value of a stimulus” (Barrett et al., 2007, p. 382). Continuing this theme, Lindquist et al. (2012 p. 124) employ *core affect* to refer to “the mental representations of bodily changes that are sometimes experienced as pleasure and displeasure with some degree of arousal,” and argue that it is related to the identification of and response to motivationally salient environmental stimuli. Representations of bodily states relies on previous experience which we may presume to rely, at least in part, on the outcome of the consequences of operant responding. Lindquist et al. (2012) concur with Panksepp (1998) that emotions fulfill a homeostatic function that indicates the value of approach/avoidance with respect to environmental stimuli.

The neurophysiological bases of *arousal* are distributed, though cortical areas and the thalamic regions whose neurons innervate cortical areas are sensitized in the course of arousing experience (LeDoux, 1998, 2000, 2003). LeDoux (1998 pp. 287–291) notes that four systems found in the brain stem are involved in arousal, each of which generates a different neurotransmitter: acetycholine (ACh), noradrenaline, dopamine, and serotonin. The amygdala, which is implicated in the production of danger signals, and the nucleus basalis, the latter a repository of ACh, are particularly relevant. Lesioning of either reduces the capacity of fear stimuli to engender arousal; stimulation of either generates cortical arousal (LeDoux, 1998, p. 289). In response to arousing stimuli, the amygdala induces the nucleus basilis to release ACh throughout the cortex. Emotional stimuli in particular produces substantial arousal (as compared with the limited arousal engendered by any novel stimulus), an observation that LeDoux ascribes to the involvement of the amygdala.

The hormones, oxytocin, and testosterone, also play a part in regulating fear and aggression as well as nurturance and affiliation. The neurotransmitter, serotonin contributes to the reduction of anxiety, so that the reduction of CNS serotonin impairs impulse control and is implicated in violence, impatience, and the assumption of risks of punishment or injury (Higley et al., 1996). The administration of serotonin by means of selective serotonin reuptake inhibitor (SSRI) medication modulates antisocial tendencies (Knutson et al., 1998). While dopamine has a general role in the anticipation of rewarded behavior, it may have a particular affinity with behavior that eventuates in (reported) arousal since it is associated with excitement, engagement, and the involved pursuit of primary reinforcers. It is, moreover, involved in energizing higher motor cortex areas on which SEEKING relies (Panksepp, 1998).

In their analyses of the role of dopamine release in learning, Berridge and Robinson (1998, 2003) refer to both a hedonic or affective outcome (denoting “liking” or pleasure) and a motivational element (suggestive of “wanting” or incentive salience). Liking is associated with opioid transmission on to GABAergic neurons in the nucleus accumbens (Winkielman et al., 2005). Wanting or incentive salience is a separate process, more likely associated with dopamine release and retention. Hence, far from being the “pleasure chemical” it has often been identified as, dopamine is neither necessary nor sufficient for “liking.” Manipulation of the dopamine system does, however, change motivated behavior by increasing instrumental responses and the consumption of rewards; incentive salience is a motivational rather than an affective component of reward that transforms neutral stimuli into compelling incentives (Robinson and Berridge, 2003; Berridge, 2004). In line with Berridge's (2000) argument that liking and wanting should be separated, Toronchuk and Ellis (2010) contrast PLEASURE which is relevant to consummatory behaviors and associated with opioid and GABA release, and Panksepp's (1998) SEEKING which is associated with dopamine release and which marks appetitive responses. This dichotomy is well-accommodated to the distinction drawn here since the wanting which is inherent in SEEKING is indicated by arousal rather than pleasure.

Dominance is an emotional response that varies as the consumer or managerial behavior setting permits a degree of autonomy or induces conformity by the number of behavioral alternatives it offers. It relates to autonomy and agency, and contrasts with submissiveness and harmoniousness (Barrett et al., 2007). Prosocial behavior and affiliation are associated with dopamine; opioids, with sociability; while the neuropeptide oxytocin increases feelings of trust (Panksepp, 2007). Both serotonin and testosterone are associated with feelings of dominance (Buss, 2004, 2005; Cummins, 2005). The relationship between dominance and the BPM resides in a tendency of consumers to report high levels of this emotional response as well as higher levels of pleasure in relation to more open settings. These are settings which offer a larger number of behavioral outcomes, and which are usually under the control of the consumer rather than an external agent like a marketer or government office. In the case of managerial behavior, dominance is also likely to be felt to an increased extent in situations that permit autonomous and multifaceted activity.

In a paper that positively reviews the evidence for a model of emotionality that includes dominance as well as pleasure and arousal, Demaree et al. (2005 p. 3) propose that “relative left- and right-frontal activation (may be) associated with feelings of dominance and submissiveness, respectively.”

Barrett et al. (2007) make a strong contribution to understanding the inter-relationships among pleasure, arousal, and dominance by proposing that arousal and dominance signify the *content* of core emotion or valence. The first of Barrett et al.'s sources of the content of valence, arousal-based content, denotes *activeness* and is revealed in self-reports of feeling active, attentive or wound-up, while unarousal, denoting *stillness*, is revealed in self-reports of feeling still, quiet and sleepy. Linking to Mehrabian and Russell's concept of arousal this active—still emotionality is an affective response to the presentation informational reinforcement. Barrett et al.'s second source of valence-content, relational content, concerns the extent of domination or submissiveness experienced in the presence of others: this social dimension of emotional reaction is redolent of the scope of the consumer's or manager's behavior setting. Finally, Barrett et al.'s situational source of content indicates the degree of novelty or unexpectedness of a situation, its contribution to or hindrance of an objective, and its consonance with norms and values. This too is suggestive of setting scope.

#### Emotional utility function

The manager, like the consumer, is assume ^4^ to maximize the combined consumption of pleasure, arousal and dominance so that his/her utility function is

(2) $${\text{U}}_{\left(\text{P},\text{A},\text{D}\right)}={\text{P}}^{\text{a}},{\text{A}}^{\text{b}},{\text{D}}^{\text{c}}$$

where U is the total amount of utility obtained by consumption of pleasure, arousal and dominance, P is the quantity of pleasure consumed, A is the quantity of arousal consumed, D is the quantity of dominance consumed, and a, b and c are empirically determined parameters such that a + b + c = 1.

#### Bilateral contingency and emotion

We assume that managers experience pleasure, arousal, and dominance as a result very largely of inputs of informational reinforcement which relate to symbolic representations of the success of market mix implementation in the market place. Sales figures and profitability manifest in pleasure insofar as they relate to the enhancement of the resource base of the enterprise; in arousal insofar as they refer to the achievement of a higher corporate reputation; and dominance insofar as they reflect greater autonomy of the firm in the capacity to meet its goals, raise capital. Over and above the specific rewards provided to managers, such as higher salaries and promotions, these corporate-level enhancements may result in managerial emotional responses. By comparison with salary and promotion, they derive relatively directly from the relationships of the firm with its customer base.

The chief medium through which managers directly receive emotional reward as a result of profitably fulfilling consumers' requirements is necessarily in the form of informational reinforcement (though if they are recompensed by bonus payments or commissions that are based on levels of sales, they also receive utilitarian reinforcement as a direct result of responding to consumer demand, and in a rationally functioning firm, they will presumably so benefit through salary adjustments and promotions in a somewhat indirect fashion). How is it possible for informational reinforcement, which we have previously identified with arousal, to give rise to all three emotions considered by Mehrabian and Russell (1974)? The version of the BPM that has thus far been the subject of this paper presents its variables in extensional terms; but there are also versions of the model that employ intentional and cognitive variables in order to continue explanation beyond that possible for the extensional portrayal of consumer choice (Foxall, 2007a).

Informational reinforcement, as it is conceptualized in the purely extensional depiction of the BPM, consists in the auditory, visual, and other sensory elements that act as reinforcers for operant behavior. In the cognitive depiction of the BPM, without making any ontological adjustments about the nature of informational reinforcement, we understanding it in terms of symbolic reinforcement which has its effect on behavior by virtue of its cognitive and affective functions. It is because we are considering, at the cognitive and affective level, informational reinforcement to be a source of symbolic reinforcement that we can conceptualize the manager's utility function in terms of utilitarian reinforcement and informational reinforcement as represented symbolically (Foxall, 2013).

### Neuroeconomic analysis

**Figure 8:**
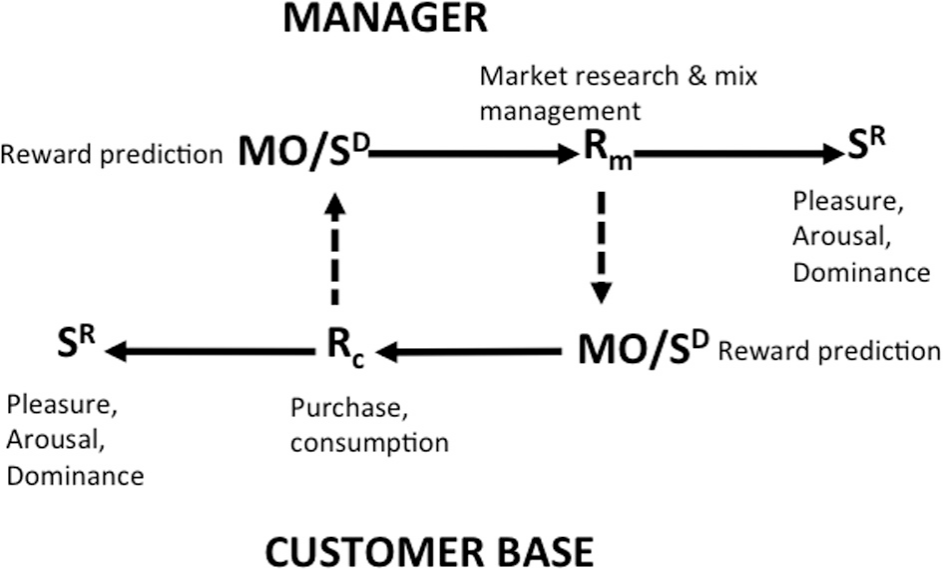
**Bilateral contingency between the marketing firm and its consumerate in terms of reward prediction error**.

The third level of bilateral contingency is that obtaining between individual marketing managers and the firm's consumerate depicted as reciprocally generating reward predictions which engender behaviors that reinforce one another's conduct (Figure 8). The ways in which the imminence of rewards is signaled to managers by the behaviors of the consumerate and vice versa may be depicted in terms of “RPEs” between the expected rewards and the actual rewards achieved (Schultz et al., 1997). These signals, which form a strong core of neuroeconomic analysis (Glimcher, 2011) are discussed in detail below after a distinction is made between two styles of neuroeconomics and their relative relevance to the analysis of bilateral contingency.

#### Modes of neuroeconomic analysis

The role of neuroeconomics in explanation requires elaboration. Ross (2008) distinguishes two styles of neuroeconomics, which he terms *behavioral economics in the scanner* and *neurocellular economics*. Behavioral economics in the scanner (BES) is depicted by Ross as stemming from the dissatisfaction of some behavioral economists with neoclassical microeconomics who, he argues, attempt to substitute psychological findings and reasoning for standard economic analysis. He argues that BES is “naively reductionist” and denies economics the right to model its subject matter abstractly, something permitted of other sciences. BES simply performs repetitions of standard behavioral-economic experiments such as the ultimatum game, the Prisoner's Dilemma, and intertemporal choice protocols used to access consumers' discounting of future outcomes during the observation of participants' brain functions via fMRI procedures. It is *neurocellular economics* that is of relevance to the current project. We can depict BES as a form of biology in the service of economics.

Neurocellular economics (NE), by contrast, is economics in the service of biology. It employs the models derived by mathematical economics, especially those of constrained maximization and equilibrium analysis, to represent brain structures and functions. The underlying assumption is that brains, like markets, are “massively distributed information-processing networks over which executive systems can exert only limited and imperfect governance.” NE is an approach to neuroeconomics that uses economic analysis to understand the neurobiology underpinning economic behavior (Glimcher, 2011). It is NE that is of primary relevance to the analysis of bilateral contingency since we are attempting here to establish the ways in which the behavior of other actors in the economic system impinge on the neuronal activity of consumers and managers respectively and prime them for the receipt of reinforcing or punishing outcomes of their own behaviors.

#### Reward prediction error

It has long been suspected, on the basis of experiments in which monkeys receive food rewards while the activity of dopaminergic neurons in the VTA is recorded (Schultz, 1992), that dopaminergic neurons code reinforcement (Robbins and Everitt, 2002). The responding of these cells to food rewards which takes place in phasic bouts is transferred, after the establishment of predictive stimuli, to those stimuli: the dopaminergic neurons respond to the CS rather than to the reward. Moreover, should the reward not appear, the activity of the dopaminergic neuron (which is recorded at the level of the individual cell) is depressed precisely when the reward was predicted to occur. As Robbins and Everitt (2002, p. 174) point out, this is indicative that the dopaminergic activity is implicated in the establishment of an internal representation of the reward (Montague et al., 1996).

RPE is the difference between a reward actually obtained and that which was predicted or expected. A negative RPE results when the reward is predicted but not obtained; a positive RPE, when a reward is not expected but is nevertheless obtained (Schultz et al., 1997). The reason why this subject has assumed such prominence in neuroeconomics is the possibility that RPEs may be reflected in dopaminergic neurons' firing rates. If so, the mechanism suggests an obvious linkage between neoclassical economics and neuroscience that is fundamental to the emerging discipline of neuroeconomics. In the present context, it adds to the explanatory power of operant psychology by proposing an underlying causal connexion (Glimcher, 2011).

While, in Pavlovian learning, the predictive significance of a signal (CS) for the arrival of a reinforcer is paramount, in operant learning, which is the principal paradigm we are using to interpret the behaviors of the marketing firm and its consumers, signals (S^D^s or MOs) influence the rate of repetition of a response that has previously led reliably to gaining the reinforcer (Schultz and Dickinson, 2000; Daw, 2013; Daw and Tobler, 2013). Associationism, which embraces both of these learning paradigms, argues that both involve the establishment of an association between the representations of either a signal (Pavlovian conditioning) or a response (operant conditioning) and the reinforcer. The procedure in which the association is formed requires that the reinforcer follow closely and reliably on the presentation of either the signal or the response, such that each repetition of the signal or response leading to the reinforcer strengthens the association (Schultz and Dickinson, 2000; see also Schultz, 2010).

The key determinant of whether a signal engenders learning, however, is not its simple presentation but its being unpredicted, novel or surprising (Di Chiara, 2002). The extent to which a stimulus is unpredicted is shown by means of a *prediction error* term, (λ − ΣV),where λ is the strength of association with the reinforcer that predicts fully the occurrence of the reinforcer, and ΣV is the combined associative strength of all signals present on the learning episode in question. The prediction error (λ − ΣV) indicates the extent to which the appearance of the reinforcer is novel, surprising, unpredicted or unexpected.

Schultz and Dickinson (2000) draw two conclusions from this which are relevant to the present discussion of bilateral contingency. The first concerns the evocation of emotions by the reinforcers and punishers resulting from operant learning, as posited by Rolls's (1999) theory of emotion. Schultz and Dickinson (2000) define learning as acquiring predictions of outcomes whether these take the form of “reward, punishment, behavioral reactions, external stimuli, internal states” (p. 476). Internal states include emotions; hence, the reinforcing stimuli that evoke emotion-feelings may also predict those feelings.

The second is Schultz and Dickinson's proposal of a sort of homeostatic principle by which behavioral outcomes that produce a mismatch (prediction error) between expected and actual reward alter subsequent behavior so as to reduce the gap between outcome and prediction. By explaining how behavior is modified in light of experience, this appears to be a mechanism for reinforcement. It explains how behavior is modified in light of experience. The process of behavior modification continues until the prediction error is zero at which point the discrepancy between expected/predicted reinforcement and actual reinforcement is eliminated. The outcome occurs exactly as predicted. This process, in line with blocking, confines learning to stimuli that predict unexpected/surprising/novel events, and eliminates learning with respect to redundant stimuli. This reasoning is very much in line with behavioral/operant learning and provides a neurophysiological explanation of learning. In instrumental or operant learning, the response manifests an expectation of reward; when the prediction is falsified by the occurrence of an unpredicted or not-fully-predicted reward (or a punisher), there is a RPE which influences future predictions and behaviors. This, of course, is the essence of operant learning. RPEs thus influence reinforcers, punishers, external signals such as attention-inducing stimuli, and behavioral goals/targets.

The import of RPEs in the current analysis is that they link consumer and managerial behaviors by showing how each relies on signals from the other as to the impending consequences of behavior; these signals may functions as discriminative stimuli or MOs for further response.

## Levels of analysis revisited

### Feasible and infeasible levels of analysis

The grounds on which both organizations and their separate members may be understood as contextual systems are as follows. Each manager's behavior is constrained by the behavior setting in which he/she works and by the pattern of reinforcement available to him/her. The manager's behavior setting scope/dominance is determined to some extent by his/her ability to manage the structure of this pattern of reinforcement and the scope of the behavior setting, and by his/her ability to influence other managers' setting scope and pattern of reinforcement. But we can also speak of the corporate behavior setting and of the pattern of reinforcement that follows from the delivery of corporate outputs (in terms of marketing mixes) to the marketplace (Vella and Foxall, 2011, 2013). The corporate behavior setting is composed of the strategic scope of the firm, predominantly its product-market matrix which defines the kind of organization it is, its purpose, the nature of its customer base and therefore the wants it is attempting to fulfill. It will also embrace the firm's overall policies, goals and objectives, and, following its resource audit, its capabilities, all of which determine the way in which it views novel opportunities and dangers as signaled by the marketplace and comparative competitive advantages. The reception its marketing mixes receive from customers determines the success of the firm and thus the extent to which its overall behavior pattern remains constant (providing similar marketing mixes) or changes (devising new or modified mixes). The two aspects are related in that success or failure in the marketplace may lead to a reassessment of the firm's scope and a consolidation of or change in its strategic direction (Foxall, 2014b).

What these examples have in common is that they relate individual human behavior, which is a personal level phenomenon, to the super-personal level of environmental contingencies which in each case are observable and measurable; the pattern or sequence of such contingencies can, therefore, be related systematically to the pattern or sequence of individual behavior; the behavior can then be presented as a function of its consequences. The result is a functional explanation of molar patterns of behavior that invokes the correlational law of effect (Baum, 1973). The behavior of the firm as a whole, i.e., the emergent generation of a marketing mix, is by definition not a personal level phenomenon. We may designate it *supra-personal* insofar as it is different from, greater than, over-and-above the combined behaviors of the members of the firm. The fortunes of the firm, as we have argued, depend on the reinforcing and punishing consequences of such behavior which in turn rely on the reception the marketing mix receives from the consumerate. Such organizational behavior depends at some level on the neurophysiological events responsible for the behavior of the firm's individual members, just as it depends on those managers' behavior being reinforced and punished by its immediate consequences that determine the success or failure of each manager. But there is no justification for ascribing a neurophysiological level of analysis to the organization. There is no way of combining or averaging the neurophysiological events of each manager to produce a composite measure that would explain the behavior of the organization. There is no bilateral contingency that links firm level behavior with that of the consumerate via meaningful neurophysiological mechanisms. All of the relevant interactions between firm and consumerate, i.e., those that predict and explain the behaviors of each, can be described at the supra-personal level (in the case of the firm) and the personal level (in the consumerate).

However, we can isolate a useful mode of explanation in terms of the emotional rewards that individual managers and individual consumers receive, each as a result of the behavior of the other. There is another useful explanatory mode at the level of neuroeconomic RPEs that result for managers from the behaviors of consumers and for consumers from those of managers. The managerial behavior that influences consumer choice may actually be that of the firm.

### A framework for research

The chief implications of the foregoing for organizational neuroscience lies in clarification of the kinds of investigation that can reasonably be conducted within this emerging framework of conceptualization and analysis. The argument is that neurophysiological explanation, in contrast to that of operant psychology, cannot be extended beyond the individual. This means that, although operant psychology may find an expression in the study of supra-individual systems such as organizations like the firm, this mode of investigation is denied to organizational neuroscience. The chief implication for the development of consumer behavior analysis and the concept of the marketing firm is that the super-personal level of analysis, in which the behavior of the firm is understood in terms of the effects that its emergent outputs (notably marketing mixes) have had on its primary environment, namely its consumerate, may be underpinned by organizational neuroscience as long as this is confined to the behavioral implications for individual managers and consumers and not abstracted to the organizational level of analysis. All of the modes of analysis advocated here can be supported by the identification of bilateral contingencies that closely link the behaviors of the transacting parties via observable and operational variables; those which have not been supported by the foregoing analysis are not realized in bilateral contingencies.

**Figure 9:**
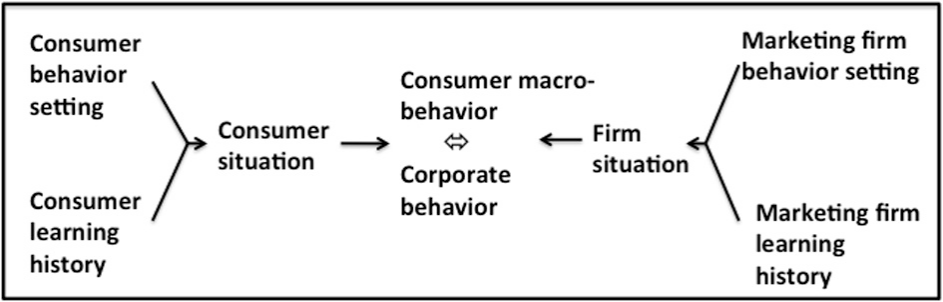
**Bilateral contingency between the marketing firm as a metacontingency and the macro-behavior of the consumerate of final buyers**.

**Figure 10:**
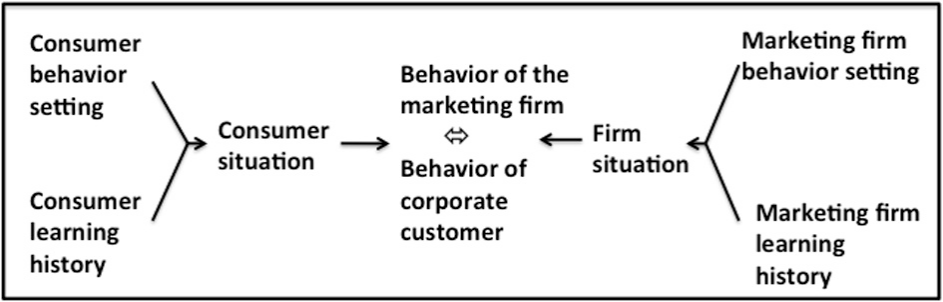
**Bilateral contingency between the marketing firm as a metacontingency and a corporate customer as a metacontingency**.

At the *supra-personal level* of exposition, firms' behaviors can be identified in terms of the marketing mix elements they introduce to the market (and these, in turn, can be traced back to their marketing intelligence procedures, their goal-formation through strategic audits of their comparative capabilities and the opportunities of the marketplace, their strategic and marketing planning, the devising and implementation of their marketing mixes). The fortunes of these marketing mixes can be ascertained through analysis of their impacts on sales and profitability. These are not easy measures to obtain in practice but attempts to secure them form part of the feedback mechanisms on which firms rely. It is feasible at this level of analysis to identify a firm-level behavior setting and learning history, and therefore a firm-level situation; the behavior attributable to this corporate situation has implications for the behavior of the firm's consumerate whether this comprises a mass of individual consumers whose collective actions amount to what Biglan and Glenn (2013) nominate macro-behavior (Figure 9) or one or more corporate customers the behavioral outputs of which can be characterized as metacontingency (Figure 10)

The equivalent of supra-contingency at the level of the individual consumer or manager is the *super-personal* level of explanation. Super-contingency refers to the control of an individual's behavior by contingencies of reinforcement, the operant conditioning paradigm exemplified by the three-term contingency. Although this level of exposition stands alone as a means of predicting individual behavior, especially in the relatively closed settings of the operant chamber, its explanatory power may be extended by considering the sub-personal, neuronal, ramifications of operant reinforcement. As the earlier discussion shows, the receipt of reinforcers is mediated by RPEs and leads to emotional reactions that reflect the pattern of reinforcement. At the super-personal level of exposition, both consumer and managerial behavior can be associated with patterns of rewards and punishments: a large body of research on the BPM has established this for consumer behavior and a far larger range of research studies have endorsed the principle for managers.

At the *personal level* of exposition, intentional idioms may be ascribed in the explanation of behavior as long as the ascription is limited so as to be consonant with empirical research findings at the super-personal and sub-personal levels (see Foxall, 2004, 2007a,b, 2013). This personal level of exposition differs from the other levels in providing an interpretation of behavior that employs intention idioms rather than the extensional language of science. That is, it proceeds in terms of beliefs and desires, emotion-feelings and perceptions that are necessary to render the behavior intelligible. Intentional exposition is used when extensional language no longer suffices to provide an understanding of behavior, principally when the stimuli responsible for behavior cannot be identified. The super-personal and sub-personal levels of exposition are integral to this personal level since they are instrumental in the creation and support of the intentional idioms that enter into personal level interpretation. Hence, in the context of neuropsychology, the *sub-personal level* of exposition involves the neurophysiological events that enter into interpretations of behavior in intentional and decision-making terms (Dennett, 1969; Foxall, 2007b,c).

**Figure 11:**
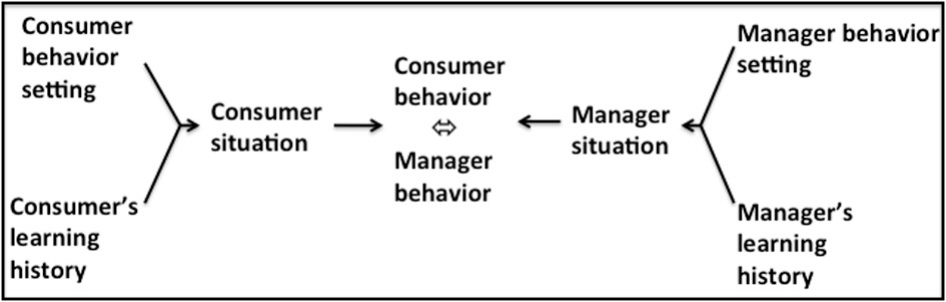
**Bilateral contingency between the individual manager and the individual consumer**.

The affect-reward and neuroeconomic levels of analysis introduced in this paper involve the super-personal, personal, and sub-personal levels of exposition. They refer to the behavior of single individuals rather than to organizational behavior (Figure 11)

## Conclusions

This paper has sought to understand the managerial mechanisms that facilitate the operation of the marketing firm by enhancing its exchange relationships with its customer base. Drawing on the TMF, it was suggested that the characterization of the parties to this bilateral transaction can be depicted as contextual systems, their behavior being explained in terms of the consequent products of the marketing firm and the customer base. The concept of bilateral contingency, which has been employed to describe the relatedness of the participants in marketing transactions to one another (Foxall, 1999a), is of value in emphasizing the interconnectedness of behavior systems that make up the marketplace. The various levels of analysis that have been considered suggest guidelines for the degree of abstraction with regard to relationships based on neurophysiological events can be justified in organizational neuroscience. The overall conclusion is that while firms and other organizations may be treated, by virtue of their generating outputs that are over and above the consequences of the behaviors of individual managers or their cumulative behavioral outputs, as contextual systems, only individual behavior may legitimately be explained by reference to a neurophysiological sub-personal level. Both individual organisms and human organizations may be treated as contextual systems but only the former constitute neurophysiological systems.

Future research on the marketing firm and bilateral contingency could usefully examine the role of the neuronal basis of cooperative behavior and trust as they are related to both intra-firm and extra-firm relationships. It would be particularly useful to understand better how trust and cooperation vary between (a) firm ⇔ firm relationships and (b) those linking the firm and final consumers.

### Conflict of interest statement

The author declares that the research was conducted in the absence of any commercial or financial relationships that could be construed as a potential conflict of interest.

## Footnotes

^1^This neologism refers simply to the totality of the firm's actual or potential customer base.

^2^Models developed in organizational sociology of the interaction of the firm and its environment in terms of strategic management are also of relevance to the market-exchange level of analysis explored below (e.g., Hannan and Freeman, 1989; Pfeffer and Salancik, 2003).

^3^The paper employs the term *reinforcer* to refer to any environmental stimulus that follows the emission of a response and which has the effect of increasing the rate at which that response is performed. This is in line with the usual meaning of a reinforcer as something that strengthens, in this case something that strengthens a response by increasing the probability of its recurrence. This usage is also consonant with the understanding of a reinforcer as something for which an organism will work to achieve. A *punisher* is a consequent stimulus that decreases that rate. Positive reinforcement involves the reception of a reinforcer; negative reinforcement, escape from or avoidance of a punisher. A punisher may also be understood, therefore, as something an organism will work to avoid or escape from. This usage accords with that of Skinner (e.g., 1974) and other radical behaviorists, though it is not followed universally. The term *reward* is employed in this paper to refer to emotional reactions that may affect the rate of behavioral performance and which are elicited by reinforcing stimuli provided by the external environment (Rolls, 1999).

^4^Whether managers' utility functions can be represented in this way remains an issue for empirical research, of course, though work in progress by the Consumer Behavior Analysis Research Group at Cardiff University and Consuma at the University of Brasilia is seeking to establish the fact of the matter for both consumers and managers. In the meantime, Equation (2) remains an assumption.
